# Determinants of willingness to accept kidney transplantation among chronic kidney disease patients in Ghana

**DOI:** 10.1186/s12882-021-02335-9

**Published:** 2021-04-13

**Authors:** V. Boima, M. B. Amissah-Arthur, E. Yorke, D. Dey, Delali Fiagbe, A. E. Yawson, J. Nonvignon, C. C. Mate-Kole

**Affiliations:** 1 Department of Medicine and Therapeutics, College of Health Sciences, University of Ghana Medical School, University of Ghana, P. O. Box 4236, Korle-Bu, Accra, Ghana; 2 Department of Psychiatry, College of Health Sciences and Center for ageing studies, University of Ghana Medical School, University of Ghana, Accra, Ghana; 3 Departments of Biostatistics, College of Health Sciences, School of Public Health, University of Ghana, Accra, Ghana; 4 Department of Psychology, College of Humanities, University of Ghana, Legon, Ghana; 5 Department of Health Policy, Planning and Management, School of Public Health, University of Ghana, Legon, Accra, Ghana

**Keywords:** Willingness, Kidney transplantation, Determinants

## Abstract

**Background:**

The burden of chronic kidney disease in Africa is three to four times higher compared to high-income countries and the cost of treatment is beyond the reach of most affected persons. The best treatment for end stage renal disease is kidney transplantation which is not available in most African countries. As kidney transplantation surgery is emerging in Ghana, this study assessed factors which could influence the willingness of patients with chronic kidney disease to accept it as a mode of treatment.

**Methods:**

This cross-sectional survey was carried out among patients with chronic kidney disease in Korle-Bu Teaching Hospital. A consecutive sampling method was used to recruit consenting patients. A structured questionnaire and standardized research instruments were used to obtain information on demographic, socio-economic characteristics, knowledge about transplantation, perception of transplantation, religiosity and spirituality. Logistic regression model was used to assess the determinants of willingness to accept a kidney transplant.

**Results:**

342 CKD patients participated in the study of which 56.7% (*n* = 194) were male. The mean age of the participants was 50.24 ± 17.08 years. The proportion of participants who were willing to accept a kidney transplant was 67.3% (95%CI: 62.0–72.2%). The factors which influenced participants’ willingness to accept this treatment included; willingness to attend a class on kidney transplantation (*p* < 0.016), willingness to donate a kidney if they had the chance (*p* < 0.005), perception that a living person could donate a kidney (*p* < 0.001) and perceived improvement in quality of life after transplantation (*p <* 0.005). The barriers for accepting kidney transplantation were anticipated complications of transplant surgery and financial constraints.

**Conclusion:**

More than two-thirds of CKD patients were willing to accept a kidney transplant and this is influenced by multiple factors. Government health agencies must consider full or partial coverage of kidney transplantation through the existing national health insurance scheme. Further, efficient educational programmes are required to improve both patients’ and physicians’ knowledge on the importance of kidney transplantation in the management of end stage renal disease in Ghana.

## Background

Chronic kidney disease (CKD) is a major public health problem especially in low and middle- income countries (LMICs), where its prevalence is higher than the global prevalence. Worldwide, prevalence of CKD is between 11 to 13% compared to 13.9% in sub-Sahara Africa (SSA) [1, 2], with a prevalence between 17% in Ghana and 30% in Zimbabwe [1]. The high prevalence of CKD among young people in this sub-region can be attributed to APOL1 renal risk alleles, which are high in frequency in SSA [3]. Importantly, in sub-Sahara Africa, including Ghana, CKD mostly affects people between 20 to 50 years of age representing the economically productive group of society, most of whom succumb to the disease because the cost of renal replacement therapy is beyond their reach [4].

The treatment options for chronic kidney diseases include haemodialysis, peritoneal dialysis and kidney transplantation, with hemodialysis being the most common treatment in sub-Saharan Africa [5]. In LMICs, treatment rates are low, leaving most patients symptomatic, due to the high cost involved, inadequate skilled personnel and limited resources [5, 6] In 2017, 661 patients were on haemodialysis [7] in Ghana and only 17 live donor transplants were performed between 2008 and 2014 [8].

Kidney transplantation is carried out in few African countries, namely South Africa, Nigeria, Mauritius and recently Ghana [9]. Although kidney transplantation is the best treatment of end stage renal disease (ESRD), transplant rates are low in Africa [9]. The challenges faced by patients tend to influence their perceptions of the disease and the available treatment options. Observations at a large tertiary facility in Ghana revealed that patients who were referred to the facility for specialist management of their CKD often presented very late. A key factor was because they sought alternative treatment options including unorthodox methods before seeking expert medical treatment [10]. A common explanation is the lack of funds to seek formal health care and unavailability of health care facilities in a financially constrained setting like Ghana. In addition, patients and their caregivers hold varying views and perceptions about chronic kidney disease and its management. At the patient level are factors relating to cost, social, traditional and cultural beliefs, religious beliefs and fear of side effects from haemodialysis [11, 12]. In addition to these factors, the acceptability of receiving a kidney transplant may be influenced by other factors including lack of knowledege of the need for kidney transplantation.

Though kidney transplantation has been available in Ghana since 2008, majority of the patients with ESRD remain on haemodialysis. The uptake of transplantation remains low and as such there is a need to assess the knowledge of patients concerning kidney transplantation and their perception and willingness to accept this as a treatment option. Identifying factors that influence the willingness to accept a kidney transplant will help health care providers tailor client education to positively impact on patients’ decision-making regarding transplantation. Furthermore, these will help policymakers to generate policies that will address the factors that negatively influence willingness to choose a transplant as treatment option. The current study, therefore, seeks to assess CKD patients’ knowledge on kidney transplantation and their willingness to accept a kidney transplant as a treatment option for ESRD.

## Methods

### Study design and setting

A cross-sectional, quantitative study that was conducted among patients diagnosed with chronic kidney disease at the medical outpatient clinic and the haemodialysis unit of the Korle-Bu Teaching Hospital (KBTH). The hospital is a tertiary facility in Accra, Ghana and offers haemodialysis and kidney transplantation treatment options for patients with kidney failure.

### Target population and sampling

Patients diagnosed with ESRD or Stage III-V CKD were recruited for the study. The study involved only adults aged at least 18 years. Participants were recruited from the medical renal outpatient clinic and the haemodialysis units of the hospital. Patients excluded from the study included those who were acutely ill or already had a kidney transplant done for them.

Each of the study participants were made to provide a written informed consent after adequate information about the study has been provided to them by trained research assistants. The dialysis patients were recruited while they were waiting for their dialysis sessions and not during the dialysis treatment.

### Sample size calculation

The Cochran’s formula was used to calculate a minimum sample size of 342 participants for the study (*n =* minimum required sample size; α = Significance level = 5%; = z-score at 95% confidence level = 1.96; *p =* proportion of patients willing to accept kidney transplant which was 66.7% [13] and e = margin of error = 0.05).

### Pre-test

Pre-testing of the questionnaire was done at a dialysis unit outside our dialysis center among 52 patients diagnosed with ESRD. This unit has similar features as the study site to establish facial and constructive validity. This led to a revision of the language and some content of the questionnaire to improve its understanding and application during the data collection process.

### Data collection

A structured questionnaire comprising 44 items was used. The questionnaire was adapted from a previous study which comprised of 40 items (including perception and knowledge of transplant and willingness to undergo a kidney transplant) [6]. In order to address the aims of the current study, this was modified and increased to 44 items due to the addition of the wealth index used to assess participants’ socio-economic status. All data were collected electronically through the use of KoboCollect toolbox. The questionnaire was administered by the interviewer in English and in the case where a participant could not understand, read nor speak the English language, the interview was carried out in a local dialect of preference. The questionnaire was administered to 342 participants, consecutively and on average took an hour to complete. The questionnaire was used to obtain data on socio-demographic characteristics such as age, gender, marital status and educational level, employment status, income, wealth index, living status, knowledge, attitude and perception relating to kidney transplant. Study participant’s social support system were also assessed. Clinical information on health status in relation to the kidney disease and any comorbidities were also obtained. Socio-economic status was assessed using the wealth index. The asset-based wealth index, which gives a composite measure of a participant’s cumulative living standard, followed the standard used for the Ghana Demographic and Health Surveys [14]. Using principal components analysis, an index was generated to place individuals on a continuous scale of relative wealth. The wealth index was then used to separate all participants into five wealth quintiles – 1–5; with 1 representing poorest and 5 representing wealthiest. Religiosity and spirituality of participants was evaluated using the Index of Core Spiritual Experiences (INSPIRIT) questionnaire. It consists of six items and rated on a 5-point Likert scale with the least number indicating the lesser experience. It has an internal consistency of 0.90 [15].

### Willingness to accept a kidney transplantation

This was assessed by asking the respondents the question, “Would you undergo a kidney transplant if you are given the chance when the time comes?” The response options were: “Yes,” “No,” and “Not sure”.

### Patient knowledge, attitude and perception

#### Knowledge

The respondents rated their level of knowledge on a 5-point scale: 1- No knowledge; 2 – Below average; 3 – Average; 4 – Above average; 5 – Well informed. This was further categorized as follows: below average (1–2 points), average (3 points) and above average (4–5 points). Additional questions were asked such as “whether the participants has heard about kidney transplantation”, “whether their physicians discussed renal transplantation with them or referred them for evaluation” and “whether they are aware of transplant centers in Ghana”.

#### Attitude

The patients’ attitude was assessed with the following questions; “Do you feel the need to know more about kidney transplantation?”, “Would you attend a class about kidney transplantation?” and “If you had the opportunity, would you donate your kidney?”

#### Perception

Perception about kidney transplantation was assessed using the question, “Do you think a living person can donate a kidney to a patient who is in need of a transplant?” Furthermore, the perception of participants was assessed by asking if kidney transplantation would affect their quality of life compared to dialysis. The response options provided were, “it will not affect the quality of life”; “it will improve the quality of life”; “it will decrease the quality of life” and “I don’t know”. These were categorized into a positive response (improved quality of life) and a negative response/don’t know. In addition, the perceived barriers to undergoing a kidney transplant among participants who were unwilling to accept a kidney transplant were assessed by asking respondents to rank the level of importance they attached to each of the barriers.

### Data analysis

The data collected was extracted from Kobotoolbox in excel format and later imported into STATA version 14.2 for data quality checks and analysis. The descriptive statistics on categorical variables were reported in the form of frequencies and percentages while that of the continuous variables were presented in terms of means and standard deviation. Chi-squared\Fishers’ exact test of independence was used to test for association between categorical independent variables and the outcome variable. Welch t-test was used to compare average age of participants who were willing to accept and those who were unwilling to accept kidney transplantation. The test of normality of continuous variables was done using the skewness and Kurtosis test. Multiple Logistic regression model as well as Poisson regression model were used to assess the effect of the various independent variables on the willingness to accept a kidney transplantation. All the statistical tests were done at 5% significance level. The results obtained from the various analyses were presented in Tables.

## Results

### Participant characteristics by willingness to accept kidney transplantation

**Fig. 1:**
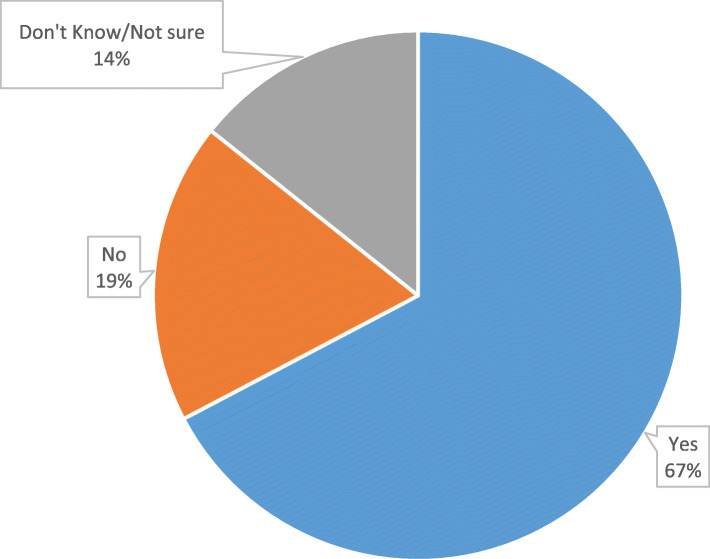
Willingness to accept kidney transplantation

In all, 342 patients participated in the study of which 56.7% (*n* = 194) were male. The average age of the participants was 50.24 ± 17.08 years. The proportion of patients *who would like to undergo a kidney transplant* was 67.3% (95%CI: 62.0–72.2%) while 18.4% were not willing to accept, and 14.3% were indecisive (Don’t Know/Not sure) about accepting a kidney transplantation (Fig. 1). Approximately 40% of the participants had completed tertiary education and 43.0% were employed. In all, 29.0% were on dialysis and the proportion of dialysis patients who were willing to undergo kidney transplant was about 12.0% higher than those who were not on dialysis (75.8% vs 63.8%, *p* = 0.028). The proportion of patients with some form of medical insurance who would like to undergo kidney transplant was 17% more than those without (69.3% vs 52.4%, *p =* 0.028) – Table 1.

**Table 1:** Participant characteristics by willingness to accept kidney transplantation

	Willing to undergo kidney transplant			
	No (%)	Yes (%)	Total(%^**c**^)	***p***-value
**Sex**				0.129
Male	57 (29.38)	137 (70.62)	194 (56.73)	
Female	55 (37.16)	93 (62.84)	148 (43.27)	
**Current Age (years)**				
Mean ± SD	51.86 ± 18.07	49.45 ± 16.56	50.24 ± 17.08	0.233
< 40 years	27 (30.34)	62 (69.66)	89 (26.02)	0.470
40-59 years	44 (30.77)	99 (69.23)	143 (41.81)	
> =60 years	41 (37.27)	69 (62.73)	110 (32.16)	
**Current Marital Status**				0.187
Married	60 (30.93)	134 (69.07)	194 (56.73)	
Single	27 (33.33)	54 (66.67)	81 (23.68)	
Widowed	20 (45.45)	24 (54.55)	44 (12.87)	
Divorced	5 (21.74)	18 (78.26)	23 (6.73)	
**Religion**				0.427
Christian	99 (31.73)	213 (68.27)	312 (91.23)	
Muslim	11 (44)	14 (56)	25 (7.31)	
Non-denominational	2 (40)	3 (60)	5 (1.46)	
**Educational Level**				0.052
No formal education	10 (52.63)	9 (47.37)	19 (5.56)	
Primary/JHS	42 (38.53)	67 (61.47)	109 (31.87)	
Senior high School/Vo	19 (25)	57 (75)	76 (22.22)	
Tertiary education	41 (29.71)	97 (70.29)	138 (40.35)	
**Employment Status**				0.359
Unemployed	41 (35.65)	74 (64.35)	115 (33.63)	
Employed	42 (28.57)	105 (71.43)	147 (42.98)	
Retired	29 (36.25)	51 (63.75)	80 (23.39)	
**Wealth quintile**				0.205
1st quintile	29 (42.03)	40 (57.97)	69 (20.18)	
2nd quintile	20 (28.99)	49 (71.01)	69 (20.18)	
3rd quintile	25 (36.23)	44 (63.77)	69 (20.18)	
4th quintile	22 (32.35)	46 (67.65)	68 (19.88)	
5th quintile	16 (23.88)	51 (76.12)	67 (19.59)	
**Social Support**				0.385
Low	40 (35.09)	74 (64.91)	114 (33.33)	
Medium	32 (27.83)	83 (72.17)	115 (33.63)	
High	40 (35.4)	73 (64.6)	113 (33.04)	
**Dialysis**				0.032*
Yes	24 (24.24)	75 (75.76)	99 (28.95)	
No	88 (36.21)	155 (63.79)	243 (71.05)	
**CKD stage**				0.086
stage 3	8 (25.81)	23 (74.19)	31 (9.06)	
stage 4	29 (46.03)	34 (53.97)	63 (18.42)	
stage 5	11 (33.33)	22 (66.67)	33 (9.65)	
End stage on dialysis	64 (29.77)	151 (70.23)	215 (62.87)	
**Has valid health insurance**				0.028*
Yes	92 (30.67)	208 (69.33)	300 (87.72)	
No	20 (47.62)	22 (52.38)	42 (12.28)	
**Preferred source of kidney**				< 0.001***
Deceased Kidney	3 (50.00)	3 (50.00)	6 (1.75)	
Living Kidney	28 (20.29)	110 (79.71)	138 (40.35)	
N/A- I don’t want a kidney	32 (94.12)	2 (5.88)	34 (9.94)	
No preference	49 (29.88)	115 (70.12)	164 (47.95)	
**Other regular Income**				0.101
Yes	2 (13.33)	13 (86.67)	15 (4.39)	
No	110 (33.64)	217 (66.36)	327 (95.61)	

*p < 0.05, **p < 0.01, ***p < 0.001, *N =* frequency; %^§^ represent row percentages, %^□^ represent column percentages; *p*-values obtained from chi-square\Fishers’ exact tests of association

### Barriers to kidney transplant among participants unwilling to accept kidney transplantation

Among the 63 participants who were not willing to accept a kidney transplant, the response rates for questions assessing perceived barriers to willingness to accept kidney transplantation was 100%. The respondents considered the following reasons as unimportant when deciding not to accept a kidney transplant- distrust for physicians (87.3%), need for more time to think and learn about it (76.2%), religious concerns (65.08%), and concern about having somebody’s organ in their body (71.43%). Further, pain and fear of surgery was not an important reason for accepting kidney transplant in 47.6% of participants. Complications of kidney transplantation was a reason for not accepting a kidney transplant in 39.7%, however, this was not an important reason for 38.1% of patients. Financial constraint was considered an important reason for not accepting a kidney transplantation in 30.2% of patients, however, it was not an important deciding factor for 49.2% of patients (Table 2).

**Table 2:** Participant’s perception towards barriers to kidney transplant among participants unwilling to accept kidney transplantation *n* = 65

	Frequency	Percentage
**I don’t trust the doctors**		
Not important	55	87.3
Somewhat important	1	1.59
Important	1	1.59
Don’t know	6	9.52
**Need more time to think and learn about it**		
Not important	48	76.19
Somewhat important	8	12.7
Important	2	3.17
Very important	1	1.59
Don’t know	4	6.35
**Religious concerns**		
Not important	41	65.08
Somewhat important	2	3.17
Important	1	1.59
Very important	6	9.52
Don’t know	13	20.63
**Complications from transplant**		
Not important	24	38.1
Somewhat important	4	6.35
Very important	25	39.68
Don’t know	10	15.87
**Surgical concerns-pain, fear**		
Not important	30	47.62
Somewhat important	3	4.76
Very important	25	39.68
Don’t know	5	7.94
**Don’t want somebody else’s organ in my body**		
Not important	45	71.43
Very important	9	14.29
Don’t know	9	14.29
**Don’t think I’ll ever need it. I feel healthy**		
Not important	54	85.71
Somewhat important	1	1.59
Important	1	1.59
Very important	5	7.94
Don’t know	2	3.17
**Financial concerns**		
Not important	31	49.21
Very important	19	30.16
Don’t know	13	20.63

### Knowledge and attitude towards transplantation and kidney donation

Most of the participants (68.7%) reported to have previously heard about kidney transplantation (Table 3). Twenty-nine percent of the total participants had discussed kidney transplantation as an alternative to dialysis with their physician; with 9.1% of the participants having ever been referred for evaluation to have a kidney transplant. About nine out of every ten participants rated their knowledge about kidney transplantation as “below average” or “average”. Approximately one-third of the participants were unaware of any transplant centers in Ghana. In addition, about two-thirds of them felt they needed to know more about kidney transplantation (73.98%).

**Table 3:** Patient knowledge, attitude and perception by willingness to accept kidney transplantation

	Willing to accept kidney transplantation			
	No	Yes	Total	***p***-value
	N(%^**§**^)	N(%^**§**^)	N(%^□^)	
**Heard about kidney transplant (Prior knowledge)**				< 0.001***
Yes	67 (26.38)	187 (73.62)	254 (74.27)	
No	44 (53.01)	39 (46.99)	83 (24.27)	
Don’t Know/Not sure	1 (20)	4 (80)	5 (1.46)	
**Physician discussed transplant with patient**				0.032*
Yes	24 (24.24)	75 (75.76)	99 (28.95)	
No	88 (36.21)	155 (63.79)	243 (71.05)	
**Ever referred for transplantevaluation**				0.936
Yes	12 (30.77)	27 (69.23)	39 (11.40)	
No	99 (33.00)	201 (67.00)	300 (87.72)	
Know/Not sure	1 (33.33)	2 (66.67)	3 (0.88)	
**Self-reported knowledgeon kidney transplant**				0.131
Below average	64 (37.87)	105 (62.13)	169 (49.42)	
Average	39 (27.27)	104 (72.73)	143 (41.81)	
Above average	9 (30)	21 (70)	30 (8.77)	
**Aware of transplant centers in Ghana**				0.187
Yes	18 (24)	57 (76)	75 (21.93)	
No	91 (35.27)	167 (64.73)	258 (75.44)	
Don’t Know/Not sure	3 (33.33)	6 (66.67)	9 (2.63)	
**Feel need to know more about Kidney transplant**				< 0.001***
Yes	58 (22.92)	195 (77.08)	253 (73.98)	
No	46 (61.33)	29 (38.67)	75 (21.93)	
Don’t Know/Not sure	8 (57.14)	6 (42.86)	14 (4.09)	
**Willing to attend a class about kidney transplant**				< 0.001***
Yes	57 (21.84)	204 (78.16)	261 (76.32)	
No	42 (66.67)	21 (33.33)	63 (18.42)	
Don’t Know/Not sure	13 (72.22)	5 (27.78)	18 (5.26)	
**Know the level of kidney function when a transplant can be done**				0.944
the kidney function	1 (33.33)	2 (66.67)	3 (0.88)	
after a patient	4 (28.57)	10 (71.43)	14 (4.09)	
I don’t know	107 (32.92)	218 (67.08)	325 (95.03)	
**Willing to donate kidneys**				< 0.001***
Yes	38 (17.76)	176 (82.24)	214 (62.57)	
No	48 (66.67)	24 (33.33)	72 (21.05)	
Not sure	26 (46.43)	30 (53.57)	56 (16.37)	
**Can a living person donate kidney**				< 0.001***
Yes	61 (21.94)	217 (78.06)	278 (81.29)	
No	7 (77.78)	2 (22.22)	9 (2.63)	
Not sure	44 (80)	11 (20)	55 (16.08)	
**Perception of quality of life after kidney transplant**				< 0.001***
Incorrect	47 (60.26)	31 (39.74)	78 (22.81)	
Correct (improve quality of life)	65 (24.62)	199 (75.38)	264 (77.19)	
**Would you ask for a kidneydonation**				< 0.001***
Yes	61 (22.18)	214 (77.82)	275 (80.41)	
No	51 (76.15)	16 (23.88)	67 (19.59)	

*p < 0.05, **p < 0.01, ***p < 0.001, *N* = frequency; %^§^ represent row percentages, %^□^ represent column percentages; *p-*values obtained from chi-square\Fishers’ exact tests of association

The proportion of participants who were willing to donate a kidney if they had a chance was 62.6% and most (81.3%) of the participants knew that a living person could donate a kidney for transplantation. The majority (80.4%) of participants reported that they would request for a kidney donation as treatment for their ESRD.

Several factors influenced the likelihood of participants to accept a kidney transplant. These were whether the *participants had heard about kidney transplantation; had discussed transplant with their physician; felt they needed to know more about kidney transplantation; were willing to attend a class on kidney transplantation; were willing to donate a kidney and who knew that a living person could donate a kidney* (*p* < 0.05) (Table 3). The majority of participants who were willing to attend a class for kidney transplantation were male between 40 to 59 years and had up to tertiary education (Table 4).

**Table 4:** Comparing age, gender, education, economic level, and health status and insurance with negative attitudes

	If there is a class about kidney transplant, would you attend		
	Yes	No	Don’t Know
	n (%)	n (%)	n (%)
**Age**			
< 40 years	70 (78.65)	14 (15.73)	5 (5.62)
40-59 years	116 (81.12)	21 (14.69)	6 (4.2)
> =60 years	75 (68.18)	28 (25.45)	7 (6.36)
**Sex**			
Male	160 (82.47)	25 (12.89)	9 (4.64)
Female	101 (68.24)	38 (25.68)	9 (6.08)
**Educational Level**			
No formal education	8 (42.11)	9 (47.37)	2 (10.53)
Primary/JHS	73 (66.97)	26 (23.85)	10 (9.17)
Senior high School/Vocationnal	59 (77.63)	12 (15.79)	5 (6.58)
Tertiary education	121 (87.68)	16 (11.59)	1 (0.72)
**Wealth quintile**			
1st quintile	43 (62.32)	17 (24.64)	9 (13.04)
2nd quintile	53 (76.81)	12 (17.39)	4 (5.8)
3rd quintile	51 (73.91)	16 (23.19)	2 (2.9)
4th quintile	63 (92.65)	4 (5.88)	1 (1.47)
5th quintile	51 (76.12)	14 (20.9)	2 (2.99)
**Insurance**			
Yes	232 (77.33)	52 (17.33)	16 (5.33)
No	29 (69.05)	11 (26.19)	2 (4.76)

*n* = frequency; %: Row percentage

### Predictors of patients’ willingness to accept kidney transplantation

The multiple binary logistic regression model revealed that *willingness to attend a class about kidney transplant; willingness to donate a kidney; perception that a living person can a donate a kidney; perception of the quality of life after undergoing a kidney transplant and whether one would ask for a kidney donation* were predictive factors of patients’ willingness to undergo a kidney transplant (*p <* 0.05). From the model, patients who were *unwilling or Don’t Know/Not sure of attending* a class about kidney transplantation had 91% (aOR: 0.09, 95% CI: 0.01–0.63) and 98% (aOR: 0.02, 95%CI: 0.001–0.37) reduced odds of accepting to undergo a kidney transplant compared to those who were willing to attend the class, respectively. Similarly, patients who were not willing or not sure of donating a kidney had 71% (aOR: 0.29, 95%CI: 0.13–0.64) and 62% (aOR: 0.38, 95% CI: 0.16–0.91) lower odds of accepting a kidney transplant compared to those who were willing to donate a kidney if they had the chance, respectively. “Not Knowing/Not sure” and “not believing” that a living person can donate a kidney was associated with 90% (aOR: 0.10 95%CI: 0.04–0.27)) and 89% (aOR: 0.11, 95%CI: 0.02–0.76) lower odds of accepting to undergo a kidney transplant compared to those who believed that a living person could donate a kidney, respectively. Having the perception that quality of life improved after having a kidney transplant was associated with about 3 times higher odds of accepting a kidney transplant compared to those who perceived that quality of life did not improve or worsened (aOR: 3.11, 95%CI: 1.42–6.78). Patients who indicated that they would be able to ask someone for a kidney if they need a kidney transplant had about 6 times higher odds of accepting to undergo kidney transplant compared to those who could not or had no one to ask (aOR: 5.82, 95%CI: 2.53–13.43) - Table 5.

**Table 5:** Determining factors of patients’ willingness to accept kidney transplant

	Sensitivity Analysis					
	Logistic regression		Penalized maximum likelihood logistic regression		Poisson regression	
	aOR (95% CI)	***P***-value	aOR (95% CI)	***P***-value	aIRR (95% CI)	***P***-value
**Heard about kidney transplant (Prior knowledge)**		0.891		0.948		0.962
Yes	1		1		1	
No	0.93 (0.38–2.28)		0.93 (0.4–2.16)		0.95 (0.66–1.39)	
Don’t Know/Not sure	1.81 (0.12–27.37)		1.36 (0.13–14.23)		1.06 (0.35–3.22)	
**Physician discussed transplant with patient,**		0.809		0.801		0.912
Yes	1		1		1	
No	1.1 (0.51–2.36)		1.1 (0.53–2.26)		1.02 (0.75–1.38)	
**Feel need to know more about Kidney transplant**		0.456		0.614		0.840
Yes	1		1		1	
No	3.18 (0.44–22.98)		2.32 (0.38–14.05)		1.19 (0.61–2.33)	
Don’t Know/Not sure	6.19 (0.18–214.6)		3.69 (0.14–96.66)		1.19 (0.36–3.97)	
**Willing to attend a class about kidney transplant**		0.016*		0.034*		0.100
Yes	1		1		1	
No	0.09 (0.01–0.63)		0.13 (0.02–0.77)		0.58 (0.27–1.24)	
Don’t Know/Not sure	0.02 (0.001–0.37)		0.03 (0.001–0.59)		0.4 (0.11–1.37)	
**Willingness to donate kidneys,**		0.004**		0.005**		0.264
Yes	1		1		1	
No	0.29 (0.13–0.64)		0.32 (0.15–0.68)		0.7 (0.45–1.11)	
Not sure	0.38 (0.16–0.91)		0.41 (0.18–0.93)		0.84 (0.56–1.25)	
**Can a living person donate kidney**		< 0.001***		< 0.001***		0.015*
Yes	1		1		1	
No	0.11 (0.02–0.76)		0.16 (0.03–0.87)		0.45 (0.11–1.87)	
Don’t Know/Not sure	0.1 (0.04–0.27)		0.13 (0.05–0.33)		0.4 (0.21–0.77)	
**Perception of quality of life after kidney transplant**		0.004**		0.007**		0.173
Incorrect	1		1		1	
Correct	3.11 (1.42–6.78)		2.76 (1.32–5.76)		1.32 (0.89–1.96)	
**Would you ask for a kidney donation**		< 0.001		< 0.001***		0.012*
No	1		1		1	
Yes	5.82 (2.53–13.43)		4.83 (2.19–10.65)		1.99 (1.16–3.42)	

*p < 0.05, **p < 0.01, ***p < 0.001, *CI* Confidence interval, *aOR* Adjusted odds ratio, *IRR* Incidence risk ratio, Background characteristics controlled for in the models were age, sex, dialysis status, insurance status, wealth quintile, religion, insurance status,

## Discussion

In this study, more than two-thirds of patients with CKD were willing to accept kidney transplantation. In particular, patients on dialysis and those who had health insurance were more likely to accept this treatment option. The proportion of our CKD population who were willing to accept a kidney transplant is similar to findings among haemodialysis patients in Saudi Arabia in a study by Alansari et al. (67.3% vs 69%, respectively) [16]. Similarly, a Nigerian study among ESRD patients showed that 66.7% were willing to accept kidney transplantation [17]. In contrast, the proportion willing to accept kidney transplantation in a study by Ilori et al. among African American population was lower. (53.9%) [6]. In another study among 239 haemodialysis patients in China, the proportion of patients willing to accept a kidney transplant was lower (46.4%) [18]. The differences observed may be attributed to variation in the study population, culture and socioeconomic characteristics.

The main factors which influenced patients’ willingness to accept this treatment were willingness to attend a class on kidney transplantation, willingness to donate a kidney if they had the chance, perception that a living person can donate a kidney and perception of improved quality of life after transplant. The important potential barriers for accepting a kidney transplantation as a treatment option were complications following transplant and financial constraints. We observed a three-fold increase in the likelihood to accept a kidney transplant among patients who indicated that quality of life improves after kidney transplantation compared to those who answered no or were unsure, showing that the benefits of perceived improvement in the quality of life after transplant plays a major role in the decision to undergo a kidney transplantation. As a result, identifying and employing approaches to create awareness about the quality of life benefits of kidney transplantation will help patients make early informed decisions leading to better outcomes.

Although, about 90% of patients with CKD rated their knowledge on kidney transplantation to be average or below average, albeit, as much as two thirds were willing to donate a kidney to someone who was in need. CKD patients who agreed to attend a class on kidney transplantation were more likely to accept kidney transplantation as a treatment option. This indicates that education will play a major role in improving their knowledge on kidney transplantation which can enhance their chances of accepting this treatment option when they develop or have ESRD.

Our study did not show a significant relationship between self-reported knowledge of kidney transplantation and willingness to accept a kidney transplant as a treatment option, however nationwide studies involving a larger sample size may be required to assess the effect of knowledge of kidney transplant on their willingness to accept this treatment among patients with CKD.

Our study also revealed that, very few patients discussed kidney transplantation as a treatment option with their physicians and less than 10% were referred by their physicians for further evaluation. These findings show that physicians either do not initiate early discussion about kidney transplantation as a treatment option for ESRD or the content of their discussions does not enable patients make informed decisions. This may be due to the busy schedules of physicians who attend to large numbers of patients and therefore do not have the time to provide effective information on renal replacement therapy options. Again, these physicians may not have the requisite knowledge to effectively educate their patients on the treatment options available. Educating physicians to inform their patients about the option of kidney transplantation will help improve patients’ willingness to undergo kidney transplantation. Again, this calls for training of nephrologists and renal nurses through existing national college programmes. Furthermore, promoting the establishment of pre-dialysis clinics which are non-existent in our setting may be a useful step.

It is interesting to note that religious concerns were unimportant to most of the CKD patients in deciding not to accept a kidney transplant which is in sharp contrast to our previous study in the community involving non-CKD patients, where religious beliefs was a strong barrier to kidney transplantation [19]. This may suggest that one’s opinion regarding a kidney transplant when they are ill and desperate for cure or treatment may be different from decisions made when they are healthy.

The findings of perceived improved quality of life post-transplant is in congruence with that of Vamos et al. where an improved self-rated health score was associated with a positive attitude towards kidney transplantation [20]. This is in line with studies where improved health after transplant and decline in health on dialysis were strong motivations for positive attitudes towards kidney transplantation [21, 22]. Ilori et al. showed that improved quality of life post-transplant is motivation for patients to accept kidney transplantation [6]. Furthermore, the low level of knowledge about transplantation observed among our CKD patient population was similar to findings by Ilori et al. [6] and Alansari et al. [16]. This is further supported by a study in a population of patients with stages 3 to 5 CKD, which revealed limited knowledge on treatment options for ESRD patients [23]. To this end, appropriate approaches to enhance positive attitudes towards willingness to accept kidney transplantation is needed. Previous studies have suggested using non-medical professionals and alternative educational resources to educate patients [24]. Among participants with progressive chronic kidney disease, Boulware et al. used social worker groups and instructions through education to improve the likelihood of undertaking living donor kidney transplantation [24].

Among CKD patients who reported their unwillingness to accept kidney transplant, a significant proportion considered complications and financial constraints as important barriers to accepting kidney transplantation as a treatment option. These findings can significantly influence their decision to accept kidney transplantation especially regarding cost of transplantation as the existing national health insurance scheme (NHIS) in Ghana does not cover renal replacement therapy. As a result, there is a need for governmental health agencies to consider subsidizing the cost of kidney transplantation in Ghana. This will promote the sustenance of the budding transplant progamme in Ghana. Furthermore, NHIS should consider full or partial support for this treatment option of ESRD in Ghana. This is particularly important because majority of affected individuals are very young and form the productive workforce of society. In addition, there is a need for education to allay the anxieties of patients regarding the complications of surgery.

Several studies have shown a significant association between socio-demographic characteristics and willingness to accept a kidney transplantation [18, 25–29]. However, we did not observe such findings, which is in keeping with findings from studies by Alansari et al. and Ilori et al. conducted among haemodialysis patients and CKD outpatients not on dialysis respectively [6, 16]. Our study did not reveal any significant association between willingness to accept a kidney transplant and mistrust for health professionals in contrast to Ilori et al’s study where mistrust for health professionals was a hindrance to accepting this treatment option [6]. This may be due to the fact that discussions about kidney transplantation are not part of routine health related discussions in our part of the world due to its limited availability. There is a need for health authorities to include issues regarding kidney transplantation in routine health discussions to highlight the need for training of health care workers on the importance of this treatment option in Ghana.

This study has the following limitation; the use of a modified questionnaire with questions drawn from different studies instead of a standardized questionnaire may affect the standard comparison of study results from other studies. However, as kidney transplantation is an emerging treatment option for patients with ESRD, such a study has utility for health and social policy. It provides information on the perceptions of a relatively young CKD population in a Low-to-Middle-Income Country (LMIC). Thus, these findings could inform future innovative interventions to improve a patient’s willingness to accept a kidney transplant.

## Conclusions

The results of the present study highlight an area that has received little attention, but is very important in understanding CKD treatment modalities in the sub-region. More than two-thirds of CKD patients were willing to accept kidney transplantation. Facilitating factors for willingness to accept a kidney transplant include willingness to attend a class on kidney transplantation, perception that a living person can donate a kidney and perception of improved quality of life after transplantation. The barriers to accepting a kidney transplant were perceptions of complications of transplant surgery and financial constraints. Improved population level educational strategies in a context where kidney transplantation is a new treatment option for patients with ESRD is imperative.

## Data Availability

Data will be made available on request. The corresponding author can be contacted via the following email address for the data; vincentboima@yahoo.com

## Abbreviations

CKD: Chronic Kidney Disease
ESRD: End Stage Renal Disease
